# Young women’s perceptions of transactional sex and sexual agency: a qualitative study in the context of rural South Africa

**DOI:** 10.1186/s12889-017-4636-6

**Published:** 2017-08-22

**Authors:** Meghna Ranganathan, Catherine MacPhail, Audrey Pettifor, Kathleen Kahn, Nomhle Khoza, Rhian Twine, Charlotte Watts, Lori Heise

**Affiliations:** 1 Department of Global Health and Development, Faculty of Public Health and Policy, The London School of Hygiene and Tropical Medicine (LSHTM), London, UK; 2 Wits Reproductive Health and HIV Institute, University of Witwatersrand, Johannesburg, South Africa; 3 MRC/Wits Rural Public Health and Health Transitions Unit (Agincourt), School of Public Health, Faculty of Health Sciences, University of the Witwatersrand, Johannesburg, South Africa; 4 School of Health & Society, University of Wollongong, Wollongong, NSW Australia; 5 Department of Epidemiology, University of North Carolina at Chapel Hill, Chapel Hill, NC USA; 6 Carolina Population Center, University of North Carolina, Chapel Hill, NC USA; 7 Umeå Centre for Global Health Research, Division of Epidemiology and Global Health, Department of Public Health and Clinical Medicine, Umeå University, Umeå, Sweden; 8 International Network for the Demographic Evaluation of Populations and Their Health (INDEPTH) Network, Accra, Ghana

**Keywords:** Transactional sex, Sexual exchange, Adolescent young women, Sexual relationships, Aspirations, Sexual agency, HIV, Sub-Saharan Africa, Risky sexual behaviours

## Abstract

**Background:**

Evidence shows that HIV prevalence among young women in sub-Saharan Africa increases almost five-fold between ages 15 and 24, with almost a quarter of young women infected by their early-to mid-20s. Transactional sex or material exchange for sex is a relationship dynamic that has been shown to have an association with HIV infection.

**Methods:**

Using five focus group discussions and 19 in-depth interviews with young women enrolled in the HPTN 068 conditional cash transfer trial (2011–2015), this qualitative study explores young women’s perceptions of transactional sex within the structural and cultural context of rural South Africa. The analysis also considers the degree to which young women perceive themselves as active agents in such relationships and whether they recognise a link between transactional sex and HIV risk.

**Results:**

Young women believe that securing their own financial resources will ultimately improve their bargaining position in their sexual relationships, and open doors to a more financially independent future. Findings suggest there is a nuanced relationship between sex, love and gifts: money has symbolic meaning, and money transfers, when framed as gifts, indicates a young woman’s value and commitment from the man. This illustrates the complexity of transactional sex; the way it is positioned in the HIV literature ignores that “exchanges” serve as fulcrums around which romantic relationships are organised. Finally, young women express agency in their choice of partner, but their agency weakens once they are in a relationship characterised by exchange, which may undermine their ability to translate perceived agency into STI and HIV risk reduction efforts.

**Conclusions:**

This research underscores the need to recognise that transactional sex is embedded in adolescent romantic relationships, but that certain aspects make young women particularly vulnerable to HIV. This is especially true in situations of restricted choice and circumscribed employment opportunities. HIV prevention educational programmes could be coupled with income generation trainings, in order to leverage youth resilience and protective skills within the confines of difficult economic and social circumstances. This would provide young women with the knowledge and means to more successfully navigate safer sexual relationships.

## Background

Young women aged 15–24 in South Africa are at high risk for HIV infection and contribute more than four-times the number of new HIV infections as do their male peers [1, 2]. A number of social, structural and biological factors have been put forward to explain the gendered nature of the HIV epidemic [1, 2]. Along with increased biological vulnerability of young women [3], relational risk factors, such as age-disparate sex, transactional relationships and experience of gender-based violence within partnerships, likely contribute to young women’s susceptibility to HIV risk [4–6]. Furthermore, individual risk behaviours—such as inconsistent condom use, higher number of partners and early age at first sex—are associated with young women’s risk of infection [3, 7]. Both relational and individual factors combined with structural factors, such as poverty and gender inequalities grounded in the socio-historical context of the country, have fuelled the HIV epidemic [4].

### Overview of transactional sex

Transactional sex or sex that is exchanged for money and/or gifts is considered to be an important contributing factor to the disproportionate prevalence of HIV/AIDS among young women in sub-Saharan Africa (SSA) [5, 6, 8]. It appears to be a common phenomenon in rural, peri-urban and urban SSA [6, 9, 10]. The concept emerged in the 1990s, as anthropologists attempted to correct the HIV field’s tendency to conflate all relationships involving exchange with formal sex work. In SSA, many non-marital, non-commercial sexual relationships involve the exchange of money or gifts: but importantly neither party in the exchange considers the relationship prostitution or sex work.

The concept of transactional sex continues to be a challenge for social scientists and the HIV field writ large [10, 11]. The challenge arises because of the tendency to assume that the categories of ‘prostitution or sex work’ and ‘transactional sex’ used in the public health literature have self-evident meanings, when in fact the meanings are ambiguous, complex and highly contested [12]. Across a number of cultures, it is common practice to exchange gifts in sexual relationships [10, 13] and expressions of romantic love can be closely linked to gift-giving [14, 15]. Cultural norms across a number of diverse cultures worldwide dictate that men should provide for women economically, an expectation institutionalised through the practice of *lobola* (bride price) before marriage in countries such as South Africa [7]. Thus, it is predominantly men who provide and women who receive material benefits in all relationships, including transactional sexual encounters [16]. Contemporary discourse on transactional sex posits that it is not formal sex work if: the exchange is undertaken within the context of a relationship (no matter how ambiguous or temporary its nature); the negotiation of the terms of the exchange is neither explicit nor upfront; and those who engage in the practice differentiate their practice from formal sex work [16]. Hence, young women engaging in transactional sex seldom identify themselves as sex workers. Their partners are referred to as boyfriends. Whereas, partners of young women who identify as sex workers are usually referred to as clients [17]. Sometimes the boundary between transactional sex and sex work can be ambiguous, particularly in short term relationships that are formed in bars. If young women practice transactional sex that is purely motivated by material gain, it can result in them transitioning into sex work for a living, if they rely on it too heavily for too long [11].

Studies that have explored women’s motivations to engage in transactional sex suggest a number of reasons. Historically, the literature emphasised that transactional sex is primarily motivated by basic survival or subsistence needs [17–19]. There is also recent evidence that indicates that young women whose opportunities are limited, may also use transactional sex to elevate their status in youth cultures that prioritise sexual success and conspicuous consumption [10, 20]. Moreover, motivations for engaging in transactional relationships are not mutually exclusive and often young women are driven by both subsistence and consumerist desires [21, 22]. Many young women also embrace romantic notions of love and security that can prompt relationships characterised by material exchange for sex; but this complexity is under explored in the SSA setting [23]. Exploring the context and motivation for transactional sex is potentially important for understanding HIV risk and HIV related risk among young women.

### Conceptual framework

Stoebenau, Heise et al. (2016) have developed a conceptual framework that captures the nuance and complexity of transactional sexual relationships and the different motivations for its practice [11]. This was done using three paradigms to describe motivations for transactional sex (sex for basic needs, sex for improved social status and sex and material expressions of love) and representing them as continua – of deprivation, agency and instrumentality – rather than as discrete paradigms. The *continuum of deprivation* describes the extent to which transactional sex is structured by poverty (absolute deprivation) as compared to economic inequality (relative deprivation). The *continuum of agency* suggests that women’s position varies over time and between relationships from the extremes of vulnerable victim to powerful agent. Lastly, the *continuum of instrumentality* is the extent to which a relationship is motivated by financial or status motivations [11].

This empirical paper aims to elucidate some of this complexity around motivations behind transactional sex by exploring young women’s perceptions of transactional sexual relationships within the structural and cultural context of rural Mpumalanga, South Africa. Specifically, the analysis considers the degree of agency perceived by young women engaged in sexual relationships and the perceived link between transactional sex and HIV infection.

## Methods

### Study context and setting

This qualitative research was embedded in an individually randomised, phase III conditional cash transfer (CCT) trial in rural South Africa (HPTN 068), details of which are published elsewhere [24]. Data collection was conducted in the sub-district of Bushbuckridge in rural Mpumalanga Province. The study setting is 500 km northeast of Johannesburg in a rural area that was part of a former homeland^1^ under apartheid, close to South Africa’s border with Mozambique. The area is poor, lacking in infrastructure and has high population density [25, 26]. Since democratic change in 1994, progress among government-led initiatives have been slow. For example, while electricity is accessible to all villages, few households can afford it for more than lighting, resulting in a half of the households reliant on wood-burning fuel. Sanitation is basic and water supply to village stand-pipes is unreliable [27, 28]. Although there is nearly universal access to primary education, quality is poor and progress often delayed [27]. There are limited employment opportunities made apparent by unemployment rates of 29% for men and 46% for women [28, 29]. This has resulted in a growing number of women who are increasingly migrating to cities for work [28, 29]. A sizeable number of people depend on a cash economy supplemented by state-sponsored, non-contributory social grants, such as the old age pension, disability and child support grants [30]. Poorer households and those that have experienced the loss of a breadwinner lack food security, with stunting being common among 20–30% of children under two years of age [31]. The most common language spoken in this area is Xitsonga and the most common ethnic identity is Tsonga, though sizeable minorities identify as Pedi, Sotho, Swazi, and Zulu [32].

The Medical Research Council (MRC)/Wits University Rural Public Health and Health Transitions Research Unit runs the Agincourt Health and Socio-Demographic Surveillance System (AHDSS) in this area and this was the platform for identifying eligible households and young women [25]. Study participants were eligible for inclusion in the main trial if they were females aged 13 to 20 years, resident in the study site; enrolled in grades 8, 9, 10 or 11 at high schools in the AHDSS study site; and had a bank or post office account or the documents available to open an account to receive the transfer. The participants were excluded if they were pregnant or married by self-report at baseline [24, 33].

### Data collection

This qualitative study used a combination of five focus group discussions (FGDs) and 19 in-depth interviews (IDIs) with young school-going women aged 18 years and above who were participants in the control arm of the main trial. Data collection was from November 2012 until March 2013. Sampling for this qualitative study was restricted to young women 18 years and above. This was due to ethical reasons associated with the need for parental assent for participants under 18 years of age. Please note that due to the time that had lapsed between baseline recruitment and this qualitative study, some of the young women turned 21 years while participating in this study. We used FGDs as they have been shown in other studies to be effective in stimulating dialogue between respondents and yielding insight into people’s thought processes about how they construct, for example, collective notions of sexual norms [34]. Overall, the FGDs were used to raise and explore broad relevant topics around young women’s relationships, which assisted in determining the structure and focus of the IDIs. Themes that emerged in the FGDs were probed further in the IDIs. There are a range of definitions of transactional sex in the literature [11, 35, 36]. For this study, we adopted the transactional sex definition advanced in the main trial (HPTN 068) in which this research is embedded [8], namely: *“a sexual relationship whereby men and women exchange sex for, or in anticipation of, material possessions or favours (such as money, clothing, transportation, school fees)”* [7, 37]. ‘Agency’ for the purpose of this analysis, is defined as *“an individual’s ability to make and enact considered choices in the pursuit of a particular end. Although possessed by the individual, agency is structured by the person’s socially shaped internality and constrained in expression by social and economic circumstances”* [21; 350].

FGDs were conducted at a village school within the study site and were facilitated by a female, Xitsonga speaker and assisted by a designated note-taker. The first author observed discussion groups with the assistance of a translator. To further ensure accuracy, the FGDs were tape recorded with participants’ permission and later translated and transcribed into English by the group facilitators. The participants were divided into three socioeconomic categories (high, medium and low) for the discussions and allocated to groups of the same SES. This was done to better explore how young women from similar economic groups discussed engagement in transactional sex. The socioeconomic variable was calculated using household monthly spending per capita data from the baseline survey associated with the main study. SES was therefore calculated as low (ranging from $1.3 to $15.4), medium (ranging from $15.5 to $32.6) and high (above $32.10) as monthly expenditures. As of 2014, the upper bound inflation-adjusted poverty line for South Africa calculated as per capita per month for food and non-food items was $45.1 [38], suggesting that even the SES category measured as high, is still much lower than the national average.

To complement the FGDs, we conducted 19 one on one, IDIs with a subset of young school-going women from the trial. Ten of the 19 participants were young women who had reported ever being sexually active and had responded positively to the question on transactional sex (*“Did you feel like you had to have sex to get gifts or money from your partner?”)* in the baseline survey of the main trial and were invited to be interviewed. The remaining nine were young women who had participated in the FGDs and were invited back to participate in the IDIs based on their levels of engagement and articulate responses during the FGDs (we had no prior knowledge of their sexual history). This allowed us to interview young women who were both sexually active and potentially not active. The IDIs were 1–1.5 h long, audio-recorded and conducted in a private location.

Themes that arose in the focus groups, but were sensitive for young women to discuss in a group setting (e.g., their sexual relationships, motivations for engaging in them and their perceptions of transactional sexual relationships) were probed further. The IDIs focused on the specific aspirations, situations and experiences of individual young women. The recordings were transcribed and translated from Xitsonga to English by the interviewer. Each transcript was quality-checked by a trained third-party researcher to make sure the translation was accurate. Data collection was iterative, with small changes made to the topic guide as the FGDs and IDIs progressed, based on a review of the data and feedback from the facilitators and interviewers.

### Participant socio-demographic characteristics

In total, 45 school-going young women above the age of 18 years participated in this qualitative study. Research participants were from rural homes in the area and half of the participants were from homesteads dependent on subsistence agriculture for survival and on state grants. Half of the participants who were dependant on subsistence agriculture were from female headed households. Seven of the 19 young women in the IDIs had small children, and two were pregnant at the time of the interview. About a third of young women interviewed in the IDIs worked in informal labour, such as domestic work, yard-cleaning or hairdressing (likely done at home or from a roadside shack). At least 12 of the 19 young women either had fathers who were working elsewhere (temporarily migrated), had passed away, or parents who were separated with the young woman living with her mother.

### Analysis

We used thematic content analysis for qualitative analysis [39, 40]. The first author familiarised herself with the content of the transcripts and created an initial coding framework based on the topic guides. The coding and analysis was done through frequent discussions between the first two authors. Coding was both deductive and inductive i.e. a coding framework was developed to guide analysis, but new codes were added as they emerged. Themes that emerged from coding of the FGDs were explored further in the IDIs. Furthermore, a content matrix was developed to display themes and sub-themes as they emerged. This content matrix was useful to help summarise and synthesise data by individual or by theme and to see emergent patterns that were cross-cutting. All data from these interviews were coded using Atlas-ti. Essentially five steps were followed in the analysis of the transcripts: (1) Data management and familiarisation; (2) identification of a coding framework; (3) displaying themes and sub-themes; (4) data reduction; and (5) interpretation [40, 41].

## Results

Four themes emerged from the analysis. First, participants gave expression to their aspirations related to education and financial autonomy; the second provides insights into the nature of young women’s sexual relationships; the third shows young women’s perceptions of transactional sex; and the final thematic element illustrates young women’s expressed agency in their sexual encounters and their perceived HIV risk. Please see Table 1 for illustrative quotes for these main themes.

**Table 1:** Main themes with illustrative quotes

Theme	Illustrative quote
Aspirations of education and financial autonomy	*“I want to complete grade 12 and go to the University and learn so I can get a job....I want to have my own money” (FGD5, low SES).* *“I want to work for them [parents], build a house and further my studies while working because [these] are the things my parents didn’t succeed on” (IDI 5, age 20 y).* *“I want to be educated so that I can able to take care of my family” (IDI 16, age 20y).*
Sexual relationships	Feelings of love: *“You won’t sleep with someone if you don’t love him; you sleep with someone if you love him” (FGD1, low SES).*
	Boyfriend’s ability to provide: *“The reason why I date the handsome guy it is just because he have money and people will take you serious and I will be famous, something like that” FGD 4, high SES.* *Like when I need money to use he was able to help me. And he was able to buy for me things like hair extensions.... I was feeling happy about it [receiving money]. IDI 18, aged 20y*
	Peer social acceptance and feeling valued: *A friend like when we are together and maybe she says my boyfriend bought me this and then I decide that I have to find a boyfriend so he can buy things for me also. IDI17, aged 20y* *“I will feel good [if he can buy gifts] and I will see how serious her is” IDI 19, aged 20y.* *“…because I want to look the same with friends”, IDI 17, aged 20y*
Subtle position of transactional sex	*I’m not ready to be paid because we never agreed upon money we just loved each other. He was able to buy for me items like hair extensions. When I wanted it, he was able to give me money to buy it. IDI 18, 20y*
Expressing agency	*‘It’s true if you have a relationship with someone he will want to sleep with you so he can do anything you want’ FGD 2, high SES.* When probed on how they would react if their boyfriends did not provide them with money or gifts: *“I would argue with him, but it would not change anything in the relationship, as I love him” IDI 9, 22y*
Constrained agency and potential HIV risk	*P1 “because nowadays young woman, if she wants something from the boy she thinks she cannot get it without having sex with that boy* *P2: It is not good because she will sleep with the person who is infected then she will get infected. FGD 4, low SES*

### Aspirations of education and financial autonomy

There appeared to be wide-spread recognition that educational achievement was the linchpin to long-term security through employment. This is despite the South African reality that education is no guarantee of employment [42]. In both the FGDs and IDIs, young women discussed the importance of school attendance and the completion of studies in order to fulfil life goals. The majority (three quarters) of young women in the IDIs said that they wanted to have secure and lucrative jobs and expressed a deep-seated need to feel financially independent. There was acknowledgement that education promotes financial autonomy through employment. This, in turn would decrease their reliance on sex as a resource to get items.

*P:.... when I want clothes and you find that I’m too old and parents are no longer taking care of me, at the end I will try to sell my body (like a sex worker), but if I am educated I will be having everything because I will be having my own money. IDI 10, aged 20y*

A theme that resonated through all the interviews was self-reliance, having access to their own money and having choices, including over their own spending decisions. A couple of young women also expressed a desire to lead a life that is better than the life lived by their parents. In addition, despite circumscribed economic opportunities in the area, young women hoped to be able to escape their current cycle of poverty. Young women indicated their intention to increase their share of disposable income and contribute towards savings for the future:

*We want good jobs - not to wash people’s clothes. If you wash people’s clothes you will not get enough money because they will give you R500 ($37.50) and R500 is a little amount. But if you have a good job you will count R500 as nothing because you will earn enough money and also be able to save it in the bank.*

*FGD 1, low SES*

Alongside employment goals, young women maintained high aspirations for their relationships and retained dreams of marriage and motherhood; two-thirds of the IDI participants expressed their desire to get married and have children. Most said they wanted this only after finishing their education and finding secure employment. At least five participants referred to leading a more ‘luxurious life’ which they referred to as residing in better quality houses or owning more expensive clothes.

The overarching narrative here is that the young women in this cohort desire a future that holds promise both in terms of financial independence and strong and loving relationships. The next section illustrates young women’s relationships and perceptions of their current reality.

### Young women’s sexual relationships in the context of rural South Africa

Most young women reported starting relationships at the age of 15–16 years and were sexually active by 17 years. Fourteen of the 19 young women participating in the IDIs reported one or more boyfriends currently. A majority of participants appeared to partner with their peers who were closer in age; the average female respondent was ~2.5 years younger than her most recent male partner. Most often young women appear to remain long-term within the same partnership, because of pregnancy, love or financial dependence on their partner. When probed on motivations for entering a relationship, young women reported becoming involved in relationships for several, often overlapping reasons, such as, satisfying feelings of love, the man’s ability to provide and social acceptance among peers.

*Feelings of love* were most often cited as reasons for starting a new relationship. Such feelings were associated with the desire for sexual intercourse and feelings of pleasure, warmth and exploration, enjoyment and having a confidante in a partner. When participants were asked in the IDIs their primary reasons for engaging in a sexual relationship and whether they felt like they had to have sex in exchange for money or gifts received, the majority (*n* = 14/19) of young women said that love was the main reason they entered into a relationship and for them engaging in sex. Most young women certainly perceived relationships involving sex to be more serious*.* When asked if they would stay in the relationships if they were not to receive money in exchange, young women were quick to respond that *“It won’t change anything and I will continue loving him”. IDI 8, aged 20y.*

*Boyfriends’ ability to provide* financial and material support was also mentioned in both FGDs and IDIs as a reason for becoming involved in relationships. In the FGDs, when asked what motivates young women to be in relationships, two-thirds of young women explicitly said that it was for money or material goods.

The remainder of young women in the FGDs were more reticent about money being their main motivation. Particularly in the IDIs, most young women were likely to back away from group consensus to say that it was love that made them engage in sexual relationships with men. Nevertheless, even though love featured in most young women’s responses, there was a clear, but implicit understanding that the need for money and gifts were important in all relationships. In their responses, however, young women did distinguish between “being opportunistic” and “feeling provided for”.

*I: Mmm… the one you had a relationship with, where did you meet him?*

*P: At school. I wanted him to help me. Like when I need money to use he was able to help me. And he was able to buy for me things like hair extensions..... I was feeling happy about it [receiving money].*

*I: Did you feel like you had to have sex with him to receive money or gifts.*

*P: No. It was the issue of love. IDI 18, aged 20y*

Furthermore, this expectation was gendered, with no intimation that men could also expect gifts from their girlfriends. Thus, along with the expectation that men should fulfil the provider role, young women appeared to harbour expectations that they would eventually receive gifts or money.

*Aaa, I won’t leave him [if he didn’t buy items that she needs or wants]. Like it can happen that I ask for something from him and he tells me that he doesn’t have money at that time, but he will buy it for me the time he will get money. I can accept and keep reminding him. He will later give me money to buy it.*

*IDI 19, aged 20y*

One young woman also mentioned feeling worried engaging in sex without money/gifts and still expected gifts even if she had sufficient money of her own.

*I: Hmm… so what would you do if he didn’t buy the items you need or want?*

*P: Hmm…I will be worried.*

*I: Would you stay with him and how would it change things for you?*

*P: Nothing will change… but I will be always worried because he didn’t buy items that I want. IDI 12, 19 y*

Some young women appeared to feel ambiguous towards money received, especially if their boyfriends’ current economic circumstances were difficult, “*I accept it [not getting gifts or money] because as a human being I know that there are times where he doesn’t have money” (IDI 8, age 20y)* and mentioned waiting for the future when he did make money. In particular, a few young women reported that if financial security comes from other sources (i.e. a brother or parents) then reliance on a boyfriend is reduced, hence relieving the pressure on the relationship.

However, a couple of young women in the IDIs openly admitted their material motivations to be in the relationship as being stronger than the romantic elements.

*P: It’s because I like things… it’s a status. He buys me airtime. I call him and friends. And boast on them that I have been bought airtime. I feel good, something like that.*

*I: What would you do if he didn’t buy you things you want like airtime?*

*P: I will just leave him, why doesn’t he buy me airtime it will mean he buy it for someone else.*

*IDI 17, aged 20y*

*Peer social acceptance and feeling valued* was mentioned by some young women who suggested that the receipt of gifts made them feel more ‘feminine’ *"he buys me things that I should have it as a girl" (IDI 8, 20y*). Furthermore, the receipt of money appeared to be used to acquire social status, with both sexes benefiting from female partners’ improved physical appearance. Young women reported using the money received to buy items such as clothes, shoes, underwear, cosmetics and body lotions that garnered peer admiration for both them and their boyfriends.

*P: It’s for status and you see I have grown up, but they don’t maintain me here at home. Or they don’t show me ways. A friend like when we are together and maybe she says my boyfriend bought me this and then I decide that I have to find a boyfriend so he can buy things for me also.IDI 17, aged 20y*

There were some other young women (*n* = 6) who appeared to value gifts for their symbolic value and the feelings associated with ‘giving and receiving’ rather than the economic and status value. Gifts were particularly appreciated when spontaneous and unexpected, rather than evolving from obligation.

*P: I feel happy [after receiving these gifts] because sometimes you find that I was not expecting it he just surprises me.*

*I: Do you feel like you had to have sex with him to receive money or gifts?*

*P: No…Sometimes he used to buy it for me; we didn’t agree that he will buy gifts for me then I sleep with him.*

*IDI 12, aged 17y*

Another young woman was keen not to give the impression to her partner that she was in the relationship for money, as *“he will think that I don’t love him. And I only want things from him”. IDI 19, aged 20y.* There was a mention of “feeling valued” in the initial stages of relationship when gifts were received, However, as the relationship progressed, there was often discontinuation of gift-giving, which was negatively attributed to familiarity.

Thus, the notion of gift giving and exchange of money and material support seemed to be so embedded and accepted in adolescent relationships that it is not visible as transactional sex, but rather as a natural component of adolescent romantic relationships.

*I: If you agree to have sex with someone are there things that you expect in return?*

*P: I’m not expecting anything as long as he remembers me when he has money*

*IDI 15, aged 20y*

An emergent theme is that as part of the courting rituals, intimate relationships are intertwined with money or material support. It appears that the rules and norms surrounding sex in exchange for money/gifts are intricate and sometimes ambiguous; gifts and money are important even in relationships characterised by love. Yet, money exchange does have one constant: a sexual relationship does not exist without the expectation of a male-to-female transfer of money or gifts. In the IDIs, most young women who have or have had a boyfriend (*n* = 17) received money and/or gifts from that boyfriend at some point during their partnership. Also, the first transfer of money or provision of a gift (usually airtime) and corresponding sexual act marks the beginning of the relationship, and, although over time the amount given may change, continued expectation of the provision of money/gifts is a significant element to sustaining the value of the relationship.

### Transactional sex or material exchange for sex occupies a subtle position within a continuum of resource exchange

**Fig. 1:**
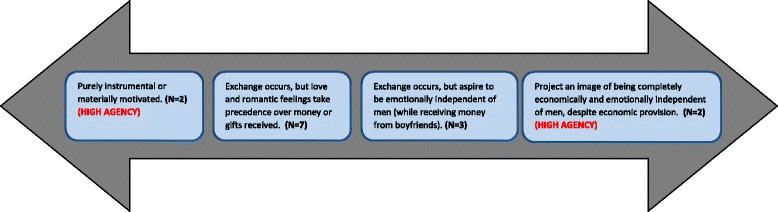
Resource exchange in young women’s relationships along a continuum

Of the fourteen young women (with boyfriends) who participated in the IDIs, ten had responded positively to the question on transactional sex in the survey *“Did you feel like you had to have sex to get gifts or money from your partner?”*. When probed on the same question during the IDIs, these young women responded ‘no’ to engaging in sex in exchange for money or gifts. From our discussions with these young women, their responses to transactional sex were more complex than their initial response to the survey question. The characteristics of exchanges ranged from purely instrumental (motivated by material gain) to motivated by emotional intimacy (organised around mutual benefits and love or romantic feelings) to seeking complete independence from men. Furthermore, an important theme that emerged from the interview responses is that young women’s perceptions around ‘sex in exchange for money and gifts’ and their rationale for engaging in an exchange-like relationship appear to be highly varied. This has been depicted in Fig. 1 to illustrate this variation.

Based on this figure, provided below are examples of the different categories of exchange-like relationships among young women.

Two young women fell into the first category of purely instrumental or materially motivated relationships. Dimikatso *(names changed to protect identities),* a young woman, aged 21 years (IDI 6), mentioned having four boyfriends, but was only in love with the father of her child. Her other three boyfriends were concurrently providing money/gifts. She was blunt about the material motivation for these three relationships, noting that if the gifts stopped: *“I won’t continue to date him and will date others”* It is clear that she would leave them if they failed to provide her with items or give her money. Her boyfriends were not older men, but rather her same-age peers. She perceived herself to be the more powerful partner in her relationships, noting, *“I would dump my boyfriend [not the father of the child], if he didn’t give me what I wanted”.* She aspires to become a social worker and get married in the future. She does not appear to have any misgivings about her arrangement with these men and seems to view gifts or provision of money as an entitlement.

The second category consisted of a proportion of young women (*n* = 7) for whom love and romantic feelings took precedence over money or gifts, although economic provision appeared to still have significance in relationships. Noma is a young woman aged 20 years (IDI 18) who reported having to borrow stationery and books from her friends for school, but felt ashamed about it. Boyfriends were a source of money for her, but she was clear that her sexual relationships with them were independent of receiving money. She equated sex with her partner with love with statements such as “*I’m not ready to be paid because we never agreed upon money we just loved each other”,* but it appears that she is also in the relationships for items with statements like *“He was able to buy for me items like hair extensions. When I wanted it, he was able to give me money to buy it”.*

The third category included young women who aspired to be emotionally independent of men, but were dependant on them for material support (*n* = 3). An example from this category is Shania, aged 20 years (IDI 17) from a family where her mother was the sole earner. Being the eldest child, she was expected to take on domestic responsibilities, while attending school. Her mother gave her an allowance for purchasing items such as school uniforms and books. She appeared to be fashion and status conscious and was aware of the latest trends popular with her peers*,* thus social acceptability was important to her, along with having extra money. Even having a boyfriend was a matter of status as indicated by, *“It’s because I like things… it’s a status*.” She accepted certain gifts from boyfriends, but without her mother’s knowledge. She expected fidelity in her relationships and would end the relationship if she were not treated appropriately. In terms of work, she aspired to become a policewoman, but also expected her partner to provide for by her. However, she was not ready to admit that she was in a relationship that had an exchange element.

*I: How do you feel about receiving airtime from your boyfriend?*

*P: Agh… I feel good, something like that. But I don’t feel that great. I only feel great when my mom bought it…*

*I: What would you do if he didn’t buy you things you want like airtime?*

*P: I will just leave him...IDI 17, aged 20y*

The fourth category included young women who were financially independent of men. For example, Rhandzile, aged 21 years (IDI 2) projected an image of independence and appeared to be status conscious with comments such as *“I will be ‘the’ person among my people and when people see me coming from there they will turn their heads and look at me”.* Her boyfriend provided her with money; however, she was clear that she was not dependent on him for resources and in fact, preferred to buy things with her own money and endeavoured to be equal in her relationships.

*P: And I don’t ask people’s belongings and I don’t depend on the boyfriend that on month end he will give me money. There is money that I receive every month.*

*I: Ok.*

*P: I am not a person who engages in a relationship because of the money. I don’t depend on the boyfriend.*

*I: … so where do you receive the money?*

*P: From government. IDI 2, aged 21y*

Overall, there was some evidence of negative attitudes towards gift-giving, largely due to potential associations with sugar daddy relationships or as such young women were viewed as “opportunistic”: *“Some young women are demeaning themselves because they go to their boyfriends aiming that they will get money” (IDI 3, 20 y).* Even if gift-giving or provision of money occurred as part of courtship, there were a group of young women who challenged it (i.e. receiving money for sex from boyfriends) for the same reason that others did not challenge it. Most young women visualised a financially independent and autonomous self in the future. Some used relationships and material exchange for sex as a means to achieve future financial independence through being able to afford items in the present.

*I: So when he leaves you when he has money, how do you feel?*

*P1: No I will never allow him to come to me because I will be educated by the time. So I won’t have a problem with him on that time because you will find that I will be educated and then I will get someone. Even if he comes back I won’t take him back.*

*FGD3, low SES*

Whereas others eschewed it, seeing it as a threat to that very same independence, as illustrated by this quote:

*I: How does having access to your own money make you feel?*

*P: I feel happy. Because no one will control me. IDI 17, aged 20y*

### Young women’s expressed agency in their sexual relationships

Some social science literature portrays young women as passive victims who simply wait for men to propose exchange for sex and then acquiesce [17, 43]. Other studies suggest that material exchange for sex is not necessarily a passive approach to attaining desired ends, but is rather a conduit through which women exercise ‘agency’ [21, 44]. Seeking exchange relationships could be viewed as agentic in situations where women’s options are constrained through limited family resources or circumscribed employment opportunities [44].

Evidence from this study suggests that young women can express agency when they select their partners and manage multiple relationships. For example, young women use criteria such as appearance, romance (“love”), status of the person and money (or ability to provide for them) as factors that guide partner choice. Receipt of money or gifts made young women feel more physically attractive and more confident (as they obtained items that contributed to their appearance) resulting in feelings of improved self-esteem, improved status in the community and feeling accepted by their peer group. In addition, money gave them disposable income to spend on items such food, clothes, shoes, underwear and cosmetics or to pay for school fees and transport. Furthermore, young women were using this life phase to ‘finance’ future independence. There were a few instances when young women (*n* = 2) felt trapped in relationships and were willing to tolerate infidelity from their boyfriends in order to feel secure.

When probed on how they would react if their boyfriends did not provide them with money or gifts, five of the 19 young women had negative responses. Their responses ranged from wanting to split up with their boyfriend with statements like *“I’d be angry with him and split up with him” (IDI 6, 22y)* or *“love him, but would break up with him” (IDI 1, 19 y)* to feeling annoyed, but recognising that love takes precedence, despite not receiving gifts to suggesting that the relationship was not working as he was unable to provide for her. Young women felt like this because of their perceived link between the receipt of gifts and their own ideas of self-worth. Yet, there appeared to be an expectation that *eventually* men will provide young women with the items they desire.

*Like it can happen that I ask for something from him and he tells me that he doesn’t have money at that time, but he will buy it for me the time he will get money. I can accept and keep reminding him. He will give me money to buy it. IDI 19, 21 y*

Furthermore, there appeared to be a normative notion that relationships always involved exchange and that if young women were not receiving money from a partner, then the money was being given to someone else, as illustrated by the following quote:

*I: Ok. If a boy can’t give you what you want, what do you do?*

*P: I shout at him....It means he gives someone else. IDI 17, aged 20y*

Therefore, in Fig. 1, agency is expressed by those young women on either side of the figure; those who choose transactional sex to get what they need and those who choose to go against the norm and stay independent of men.

On one end, despite receiving support from their boyfriends, some young women were able to take the initiative and end the relationship with their boyfriends if they felt mistreated; *“He was bullying me. And I don’t like that kind of a person”*. There was also mention of discontinuing the relationship if gifts were not received particularly when an expectation of receiving gifts was already in place:

*I: What would you do if he didn’t buy you the items you need or want?*

*P: I will be angry...I will date others.*

*I: How would it change things for you if he doesn’t buy you these things?*

*P: Why at first he was doing it and now he don’t. IDI 6, age 22 y*

At the other end, a smaller number of young women were clear that the expectation and receipt of gifts was part of love and courtship and that they were not dependent on their partners. This was derived from an understanding of sex as a valuable resource:

*I: Ok. What would you do if he didn’t buy you items you want?*

*P: As I’ve said…I don’t depend on the boyfriend that he will give me the money. And I don’t care about boyfriend’s money and he also knows that I didn’t fall in love with him because I am desperate. I have my own money and it is enough for me. IDI 2, 21y*

There was a difference between the FGDs and the IDIs in how young women responded to questions around transactional sex. In almost all the FGDs, participants mentioned young women using their sexuality as a resource in order to obtain items they desire or need and were thus in control of their choices. In a group setting, the general claim was that young women from higher income households were in a position to choose their partners and were able to determine whether they wanted to be with someone, irrespective of their economic and family circumstances. Thus, they could negotiate the terms of the relationship. However, some other participants believed that men were the ones who exerted control and were emotionally exploitative, as they were financially better placed even when the young woman is from a relatively richer household.

*P3: You want to know who has power between a man and a woman.*

*I: Yes, in their relationships.*

*P3: Oh! I will say is a man because a woman begs…*

*I: Mmm…how?*

*P3: Because you want money. FGD5, high SES*

Still, most FGD participants were of the opinion that sex for gifts or money made them feel like they were in charge as no one else had made the decision for them: *“It’s true if you have a relationship with someone he will want to sleep with you so he can do anything you want” (FGD 2, high SES).* In addition, there was mutual benefit in the exchange i.e. the man’s desire to obtain sex from the young woman, will make him provide whatever she wants or needs as items, in order to satisfy her.

However, the impression that young women were in control when sex was exchanged for money was expressed less emphatically in the IDIs. In almost all the IDIs, the young women understood the question, *“Some people say that young women are taking control of their lives by using sex to get what they want – what do you think?”* to imply sex with sugar daddies or older men. Even after clarification that it could also be a same age boyfriend, most young women continued to have the opinion that the concept of sex in exchange for material support with older partners was demeaning to them.

*P5: Yes you demean yourself like when you want something and your parent don’t give you and then you decide that it’s better to seek relationships with older men so they can help you .....and then that old man will sleep with you and then when you go to him tomorrow you find that he don’t care about you anymore… meanwhile he has infected you with HIV/AIDS. And then you will be sick where it will be impossible to go to him or sleep with others.*

*FGD 3, low SES*

#### Constrained agency and potential HIV risk

Whilst young women appear to have considerable agency at the point of choosing partners, once the choice is made, their bargaining power within the relationship appeared to weaken. This is particularly so when it came to negotiating condom use. For some young women, non-condom use in the relationship could be due to their restricted ability to request condoms during sexual acts, especially if there had been exchange of money.

*P1: Yes because some men can say if you sleep with me I will give you R1000 [$75] and when she hear that she agrees.*

*I: Mmm…do you think that they might use condom or not?*

*P2: Most of the boys don’t agree to use condom and when you tell him that we have to use a condom he say I don’t use condom. And he find that I already taken his money and then he will say ‘I won’t sleep with you using a condom, what were you thinking when you take my money’... FGD3, high SES*

Thus, the room for negotiation related to condom use can be difficult or it appeared that young women have to bargain to practice safe sex. Young women seem too willing to please their boyfriends, hence compromising their own needs (to use condoms) or are concerned that they would have to return their gifts if they request condoms during sex.

*I: Does it change your sexual behaviour with him like don’t use a condom?*

*P: No, sometimes we do use condoms and sometimes not.*

*I: Mmm…but it is because he gives you gifts when you don’t use a condom or is something else?*

*P: No is just that I feel happy when I see the gifts or maybe say we don’t have to use a condom and maybe at the end I win and use a condom. IDI 1, aged 19y*

There was awareness though that in such relationships (sex for money or sex for gifts), young women might be placed in a situation of limited choice and negotiating power with regard to protected sex, as men who are providing them with these items are more likely to be in control. Hence, young women might be made vulnerable to HIV, if the necessary precautions were not taken.

*P1: Yes is correct because nowadays young woman, if she wants something from the boy she thinks she cannot get it without having sex with that boy*

*P3: He will tell you that he cannot do this and this for you because he doesn’t trust you so you must have sex with him... if your situation is not good you will accept it. If situation at home is good, she will wait for her parents until the right time then they will do it for her.*

*P2: It is not good because she will sleep with the person who is infected then she will get infected. FGD 4, low SES*

Also, according to a couple of young women, there was a perception among males that if the young woman insists on condom use, she is HIV infected, thus making it difficult for young women to express their desires at the risk of appearing untrustworthy to their partners.

*I: Did you protect yourself when you started to make sex?*

*P: Yes, we were using a Choice [condom] and when time goes by, he refuses to use condoms.*

*I: When did you fall pregnant?*

*P: I was 15 years old, at first we used a Choice [condom] and he told me that it means I am taking him as he’s sick and then we went for blood test and then told us that we are not sick and then we decided to stop using condoms...Then I fall pregnant. IDI 1, aged 19y*

Overall, young women appear to express agency when initiating and terminating relationships both in terms of the choice of partner and criteria they use to select partners. Non-condom use is a risk factor for HIV in any sexual encounter, irrespective of whether it is considered a transactional sexual encounter. However, what appears to make transactional sex risky is that it places young women in a place of limited choice or constrained agency with regard to negotiating protected sex.

## Discussion

This qualitative study adds to the growing body of research around transactional sexual relationships by providing insights into how sexual relationships are negotiated among a specific group of school-going adolescents in rural South Africa. Specifically, this paper attempts to elucidate the complexities of transactional sexual relationships among young women in this particular social and cultural context. The evidence from focus group discussions and in-depth interviews conducted in rural Mpumalanga province, South Africa suggests that material exchange for sex was common among young women in non-marital relationships. There are three main findings that emerge from this research.

First, young women have a deep-seated desire to feel economically independent from their families. Similar to research on youth growing up in comparable resource-constrained settings, young women appear to have shaped their aspirations in ways that account for the limited job opportunities present [45]. The value they placed on education was shaped to some extent by the potential for financial independence. This is similar to research in higher income settings, such as from the United Kingdom and Australia which showed that adolescents are strategic in their approach towards education and view it as an accomplishment leading to long term security through employment [46, 47]. The majority of young women aspire to get rewarding jobs and live a better life than their parents. Research among adolescents in other resource-constrained settings has shown that secondary schooling is also an educational stage when young people perceive their likely academic attainment and thus form tentative ideas about when they can exit the educational system [45]. Furthermore, in this data, even if the immediate opportunities are lacking, most look outside the area for work, and many come to realise that it is necessary to move to cities or towns in order to find work. Thus, education in this context appears to be seen as promoting social mobility.

Second, the link between sex and receipt of money or gifts is nuanced and a normal part of courtship among adolescents in this setting. This sample of young sexually active women appear to partner mostly with others of similar age; their motivations include acquiring social status, improving their self-esteem, finding suitable long-term partners and gaining sexual companionship, rather than being motivated exclusively by material gain. Material gain is, however a central concept to romantic relationships. IDIs demonstrate that exchange practices are driven by young women’s need for resources; many are poor and appreciate a material benefit gained from having sexual relationships. So, lack of alternative sources of income drive young women to engage in sex for money, as has been similarly shown in data from Madagascar [48]. For some young women, the money received through sexual exchange helps fulfil their consumerist desires, which is rooted within cultural and economic processes of globalisation [20], as well as to provide them with a sense of independence, feelings of self-worth and improved self-esteem and to develop an identity within their peer network [49]. There is an important link with educational achievement here, as secondary school is a time when young girls develop impressions about their own ability vis-a-vis their peers [50]. Female adolescents in particular are socialised into relational (whether with peers, boyfriends or family) rather than autonomous roles and this makes them vulnerable to the actions and behaviours of others [51]. As adolescence is a vulnerable developmental stage, relationships have the potential to undermine confidence and self-esteem among young women [52]. Hence transactional sexual relationships are not solely about survival or consumerism, but built into complex ways in which young women define themselves in global economies. Yet, such money transfers seem to take on a deeper meaning for most young women in this study. Young women interpret receipt of money from their partners as loving or romantic gestures and as an indication of how much they are valued by their partners. Thus, money exchange is an implicitly understood reciprocal obligation that both forms and sustains sexual relationships; all such partnerships are characterised by male-to-female money transfers whether these partnerships are casual or more enduring.

The findings illustrate the complexity of transactional sex and the way it is positioned in the literature. This suggests that the term ‘transactional sex’ and the associated definition of the practice commonly used within the field of HIV is too blunt an instrument to be used to categorise different relationships, where money exchange takes place not always in the context of sex, and where even though money may be exchanged, a primary motivation for the relationship is also love. Our findings show that alongside being a resource, the provision of money or gifts has an important symbolic meaning: where money transfers, as gifts, indicate a young woman’s value and shows the commitment of the man. A sudden reduction in the amount given may show her lack of worth to the man and also her perceived lack of worth in broader society, and, in all likelihood, could be the end of the relationship. This finding lends support to similar research done in Malawi [23], urban Johannesburg [53] and Uganda [15] where the sub-text of exchange that underlies sexual relationships is not overt and negotiations that take place were not explicit. Contrary to this, research from both Zimbabwe [54] and Tanzania [55] suggests that young women actively used their sexuality as an economic resource, and often willingly entered into relationships primarily for economic gain [54, 55].

Our data show that young women do not perceive an expectation of sex from the partner’s side when gifts or money are provided. However, participants reported engaging in sex when provided resources because it is their way of reciprocating their love and appreciation for their boyfriend. This sometimes stems from a feeling of obligation, but not in all cases. Nevertheless, young women need to maintain a balance and not appear to be opportunistic and only driven by the promise of receiving gifts or money. This echoes the work of Nyanzi et al., among young adolescent school-going women in Uganda, where if young women appear to be too interested in money they may be stigmatised as ‘loose’. On the other hand, if they are not interested in money at all they may be suspected of being infected and wanting to spread HIV [56].

In this study, young women’s interdependence on their boyfriends or partners and their rationale for engaging in an exchange-like relationship was varied in terms of the value attached to it by participants: from purely transactional or personal gain-focussed to interdependence in the relationship (which is organised around mutual benefits and love or romantic feelings) to complete independence from men. But, ultimately this shows that exchanges serve as points around which romantic relationships are organised.

Third, transactional sex is one of the few aspects of youth sexual behaviours that features female agency. This is because it could be viewed as a situation in which young women are frequently positioned as actively seeking desired ends [21]. A dominant discourse in many interviews from this sample indicates that young women had considerable decision-making control over the process of relationship formation and termination, something referred to as temporal agency [57] and they used it to their advantage. They are able to select the type of partners they want, and determine when to end partnerships, hence taking some control over their own sexuality, which then has implications for HIV risk. It is important to recognise though that almost none of the young women in this sample mentioned being stuck in abusive relationships, as a result of material dependence. Young women usually assessed the attributes of potential partners in some combined form: attractiveness, peer group behaviour and money were all considered when deciding whether to acquire a partner and with whom to partner. Agency is demonstrated at both ends of the relationship continuum in Fig. 1; among those who choose transactional sex to get what they need now and those who choose to go against the norm and stay independent of men.

However, for most young women there was an (implicit) expectation of being provided for by their partners. As Luke et al. (2012) demonstrated in Kisumu, Kenya, there was evidence of an association between the value of cash received by young women and their engagement in sex, including the number of sex acts, and inconsistent condom use, even after adjusting for indicators of love and commitment. Luke et al.’s research shows that (on average) money and material support take on a transactional role in premarital relationships in the first few months irrespective of the initial motivation (i.e. love or materialistic aspiration of the young woman). Ultimately, if young women view their partners as providers and are dependent on them for income (given limited opportunities otherwise) they run the risk of being in the weaker position when it comes to bargaining for safer sexual behaviours.

Thus, even though young women expressed agency in choice of partners, their agency appears weakened once they were in a relationship and this rarely translated into decisions about HIV risk reduction or pregnancy prevention. As relationships developed, bargaining power tended to increase for the male partner. Wamoyi et al. in ethnographic research in Tanzania show that more money or gifts were necessary to seduce a young woman into starting a sexual relationship than to have sex on subsequent occasions [55]. In our study, young women complained that their boyfriends stopped giving gifts once they were co-habiting – something they attributed to familiarity and availability. Also, as ‘trust’ is built within relationships, evidence shows that condom use becomes irregular [58, 59]. This data shows that young women are aware of the consequences of not using condoms, but submit to their boyfriend’s demands to not use condoms for fear of their boyfriend leaving them or of creating an impression that they themselves are HIV infected. This reflects findings from MacPhail and Campbell’s research on South African youth and condom use, where trust in the partner is given as a dominant reason for not using condoms; however this trust is seldom based on a negative HIV test or discussion about sexual histories [60].

In terms of the association between transactional sex and young women’s vulnerability to HIV infection, the findings suggest that young women’s desires to feel independent, successful and to fulfil their needs and wants, places them in a non-negotiable position vis-a-vis their partners, who by virtue of being the provider, is in a more dominant position in terms of bargaining power. Despite having romantic notions of relationships, clearly underlying this is the need to be looked after by the man, in the form of money or material support. This makes the balance between the emotional and transactional elements in a relationship blurred. Adolescent relationships in this context are normatively transactional despite their emotional attachments and lie on a continuum in terms of levels of dependence on their boyfriends. Gift-giving and material exchange are an inherent component of relationships and in demonstrations of young women’s worth and the strength of their relationships. Given that transactional elements exist within such relationships, avenues for HIV might exist through the potential for unprotected sex. Furthermore, young women’s eagerness to please their partners put them in a position where they might yield to anything and are then placed in a non-negotiable position because of their reliance on partners. All these factors combined result in young women being vulnerable to HIV infection.

A significant limitation of this data is that men’s perspectives were not accounted for in this study. Getting men’s perspectives around gift giving, the expectations or obligations they might feel as ‘providers’, as well as their opinions on transactional sexual encounters, would have vastly enriched this data. The second is that as the sample for the qualitative data was restricted to rural young women who were 18 years and above and enrolled in school, it limits the generalisability of the data to settings with other non-school going young women and under 18 year olds. Third, an eligibility criteria for being part of the main trial was that young women and their parents/guardians had to have documentation to open a bank account. People without documentation are likely to be quite poor because identity documents are required to apply for most social services and welfare grants in South Africa. This suggests that poorer young women who have limited access to basic services and social grants in South Africa could not enrol in the study. It may be that transactional sex in this subset of disadvantaged young women is less consumerist oriented and more subsistence-driven. Hence the applicability of the results needs to be interpreted accordingly. Finally, we need to consider the discrepancy in young women’s responses to the question on transactional sex (*“Did you feel like you had to have sex to get gifts or money from your partner?”)* between the survey and the IDIs when interpreting the results. Possible explanations include young women’s interpretation of the intent of the original quantitative questions and social desirability bias or concerns young women might have about admitting to a stigmatised materialistic motivation in the IDIs. In terms of differences in responses between the FGDs and IDIs, there is a perceived sense of freedom in the FGDs where one has to speak generally about an issue. In the FGDs, young women were asked to respond to questions generally (not relating it back to personal anecdotes), whereas in the IDIs, young women were asked questions related to their personal situations. Hence, in almost all the FGDs, participants mentioned young women using their sexuality as a resource in order to obtain items they desire or need and were thus in control of their choices. Whereas in the IDIs, young women appeared more reluctant to openly acknowledge that sex in exchange for money or gifts was a conduit by which they themselves might express their sexuality and feel in control.

## Conclusion

Overall, the findings show there is recognition among young women that securing their own financial resources will ultimately improve their bargaining position in their romantic or sexual relationships, as well as open doors to an independent future. However, they appear to be caught, largely unconsciously, in a transition of making high risk immediate choices to facilitate future independence and develop self-reliance. This research underscores the need to recognise that transactional sex is socially embedded in adolescent romantic relationships, but certain aspects of it make young women vulnerable to HIV, without them explicitly recognising this risk. This is especially in situations of restricted choice and circumscribed employment opportunities. HIV prevention educational programmes should explore and evaluate models where there is coupling with income generation trainings, in order to perhaps leverage youth resilience and protective skills within the confines of difficult economic and social circumstances. This might provide young women with the knowledge and means to successfully navigate safer sexual relationships.

## Abbreviations

AHDSS: Agincourt Health and Socio-Demographic Surveillance System
CCT: conditional cash transfers
FGD: focus group discussions
HIV: Human Immuno-Deficiency Virus
HPTN: HIV Prevention Trials Network
IDI: in-depth interviews
LSHTM: London School of Hygiene and Tropical Medicine
STI: sexually transmitted infections

## Footnotes

1 Homelands were created during the apartheid regime to relegate the majority of black African people to rural enclaves.
